# Long-Term Association Between Maternal Preconception Hemoglobin Concentration, Anemia, and Child Health and Development in Vietnam

**DOI:** 10.1016/j.tjnut.2023.03.015

**Published:** 2023-03-15

**Authors:** Melissa F. Young, Phuong Nguyen, Lan Mai Tran, Long Quynh Khuong, Reynaldo Martorell, Usha Ramakrishnan

**Affiliations:** 1 Hubert Department of Global Health, Rollins School of Public Health, Emory University, Atlanta, GA, United States; 2 Poverty, Health and Nutrition Division, International Food Policy Research Institute, Washington, DC, United States; 3 Thai Nguyen University of Pharmacy and Medicine, Thai Nguyen, Vietnam; 4 Hanoi School of Public Health, Hanoi, Vietnam

**Keywords:** hemoglobin, preconception, anemia, child development, birth outcomes

## Abstract

**Background:**

The long-term association between preconception maternal hemoglobin (Hb) concentrations and child health and development is unclear.

**Objectives:**

We examined associations between maternal preconception Hb concentrations and anemia with *1*) birth outcomes (weight, length, preterm, gestational age, small for gestational age); *2*) child Hb at 3 mo, 6 mo, 12 mo, and 24 mo; and *3*) motor and mental development at 12 mo and 24 mo (Bayley scales for infant development) and cognitive functioning at 6–7 y (Wechsler Intelligence Scale for Children).

**Methods:**

We used data from a randomized controlled trial (PRECONCEPT) conducted in Vietnam. Over 5000 women who were intending to conceive were recruited, and offspring were prospectively followed from birth (*n* = 1599) through 6–7 y (*n* = 1318). Multivariable linear and logistic regressions were used to assess the association between preconception Hb or anemia (Hb < 12g/dL) on child health and development outcomes, adjusted by supplementation group (tested for interactions) and confounding at maternal, child, and household levels.

**Results:**

At preconception enrollment, 20% of the women were anemic. Maternal preconception Hb was positively associated with child Hb at 3 mo (0.06; 95% CI: 0.01, 0.12), 6 mo (0.08; 95% CI: 0.03, 0.13), 12 mo (0.10; 95% CI: 0.04, 0.15), and 24 mo (0.07; 95% CI: 0.02, 0.12). Likewise, maternal preconception Hb was associated with reduced risk of child anemia at 6 mo (0.89; 95% CI: 0.81, 0.98), 12 mo (0.81; 95% CI: 0.74, 0.89), and 24 mo (0.87; 95% CI: 0.79, 0.95). Maternal preconception anemia was negatively associated with cognition (−1.64; 95% CI: −3.09, −0.19) and language development (−1.61; 95% CI: −3.20, −0.03) at 24 mo. Preconception Hb was not associated with birth outcomes or cognitive outcomes at 6–7 y.

**Conclusions:**

Maternal preconception Hb was associated with child Hb across the first 1000 d of life. However, preconception Hb was not a significant predictor of birth outcomes or cognitive outcomes at 6–7 y in this cohort from Vietnam.

**Clinical Trial Registration:**

PRECONCEPT study (NCT: 01665378).

## Introduction

Globally anemia impacts over 571 million women of reproductive age, 32 million pregnant women, and 269 million children [1]. Anemia during pregnancy has been recognized as a key contributor to maternal mortality, poor birth outcomes, especially preterm births and small for gestational age (SGA), and subsequent child health and development [[2], [3], [4], [5]]. It is also estimated that 200 million children across the globe fail to reach their developmental potential due to inadequate nutrition and stimulation [6,7]. Further, iron deficiency anemia during early childhood has been identified as an important risk factor that adversely affects childhood development especially in developing countries [[7], [8], [9]].

Emerging evidence indicates the timing of maternal anemia during pregnancy may have differential implications for the infant [10]; however, the research to date has tended to focus on the consequences of anemia during pregnancy rather than preconception. There is a growing recognition of the importance of adolescent and preconception maternal nutrition [[11], [12], [13], [14]]. Evidence from animal and observational studies indicates that the nutritional and health status of women as they enter pregnancy can influence placental development and nutrient availability for fetal growth and development [12,15]. However, few studies have examined the relationship between preconception hemoglobin (Hb) and birth outcomes. A systematic review from 2018 [5] identified only 4 studies that assessed the association between preconception anemia (Hb < 12g/dL) and increased risk of adverse birth outcomes [[16], [17], [18], [19]]. Furthermore, data were limited for other outcomes, and the relationship with functional outcomes such as child development remains unclear [5,20].

We are in a unique position to address this important research gap, using data from a large micronutrient supplementation trial (PRECONCEPT, NCT01665378) that was conducted in Vietnam [21]. Women were randomly assigned to receive weekly preconception supplements containing multiple micronutrients (MM), iron-folate (IFA), or folic acid (FA) and then all women received daily prenatal IFA supplements [22]. Although there were no differences in birth size by treatment group [22], there were modest improvements in child development at age 2 and 6–7 y in the MM and IFA groups compared with FA only [23,24]. Our current objective was to examine the associations between baseline maternal preconception Hb concentrations (primary exposure) and anemia with child health and development from birth through 6–7 y, namely, *1*) birth outcomes (birth weight, birth length, preterm, gestational age, or SGA); *2*) child Hb at 3 mo, 6 mo, 12 mo, and 24 mo; and *3*) motor and mental development at 12 mo and 24 mo and cognitive functioning at 6–7 y. We hypothesize that maternal preconception Hb concentrations will be positively associated with child health and development outcomes across early childhood.

## Methods

### Study design, participants, and setting

Children in this study are offspring of women who participated in the PRECONCEPT study (NCT: 01665378). Details of the PRECONCEPT study have been published previously [21]. Briefly, the study was a randomized controlled efficacy trial that included 5011 women of reproductive age who were assigned randomly to receive weekly supplements containing either 2800 μg FA, 60 mg iron and FA (IFA), or MM containing the same amount of IFA, from baseline until conception, followed by daily prenatal supplements containing 60 mg iron and 400 μg FA for all 3 groups until delivery. Prior reports have documented high compliance, with nearly 80% of participants consuming >80% of the supplements both before and during pregnancy [25]. Women were followed prospectively to identify pregnancies and evaluate birth outcomes, and the offspring were followed up through age 6–7 y [24]. From 2012 to 2014, a total of 1813 women conceived, and 1589 had live births (1599 births, including 10 twins).

### Outcome measures

The key outcomes of interest include *1*) birth outcomes, *2*) offspring Hb concentration during the first 1000 d, and *3*) child development at 12 mo, 24 mo, and 6–7 y.

#### Birth outcomes

Birth weight was measured as early as possible, within 7 d after birth, using electronic weighing scales precise to 10 g. Birth length was also measured using standard procedures [26] with collapsible length boards, which were precise to 1 mm. Gestational age was calculated as the number of days between the first day of the last menstrual period (obtained prospectively by village health workers during their biweekly home visits) and the day of delivery. A preterm birth was defined as a birth occurring before 37 completed weeks of pregnancy. SGA was defined as a birth weight below the 10th percentile for gestational age and sex [27].

#### Offspring Hb concentration during the first 1000 d

Offspring Hb concentration was measured from finger prick capillary blood samples at 3 mo, 6 mo, 12 mo, and 24 mo of age using a portable field B-Hb Analyzer (HemoCue 301) (23). Child anemia was defined as a Hb value <11 g/dL [28].

#### Child development

Child development at 12 mo and 24 mo of age was assessed using the Bayley Scales of Infant Development (BSID) III [29], which includes cognitive, language, and motor subscales. The BSID-III has been translated and adapted in Vietnam using standardized methods and has been used in previous studies in Vietnam [30,31]. The raw summary scores for each of the domains were then transformed into standardized composite scores (approximately mean ± SD: 100 ± 15) to facilitate comparisons across domains. The BSID-III was administered in a quiet room at community health centers by well-trained researchers. Data quality was assessed based on weekly field-based supervision and monthly staff meetings. Site visits were also carried out regularly by study investigators, and refresher training sessions were conducted every 6 mo after the initial training to ensure testing was conducted in a standardized manner [24].

Child intellectual development at 6–7 y was assessed using the Wechsler Intelligence Scale for Children—Fourth Edition (WISC-IV) [32]. The WISC-IV consists of 4 specific cognitive domains [verbal comprehension index, perceptual reasoning index, working memory index (WMI), and processing speed index (PSI) and the full-scale intelligence quotient FSIQ)]. The WISC-IV has been translated, adapted, and validated to be a standardized test in Vietnam [33]. The WISC-IV was administered by trained pediatricians or research assistants with a master’s degree in public health. Quality control measures were emphasized in field-based supervision, monthly staff meetings, and refresher training.

### Exposure variables

The primary exposure variable was preconception Hb concentration, which was measured at recruitment and prior to receiving supplements by HemoCue 301 from a capillary blood sample obtained by finger prick [34] using standardized training protocols [35]. In the secondary analysis, maternal anemia was defined as a Hb concentration <12 g/dL [28]. In addition, an exploratory posthoc analysis was conducted using a lower Hb concentration cut-off (Hb < 10.81g/dL), as suggested by a recent pooled multicountry analysis of the fifth percentile of Hb values among healthy nonpregnant women [36].

### Confounders

Confounding variables were considered at child, maternal, and household levels. These included child age and sex, infant and young child feeding practices, childhood illnesses (fever, diarrhea in the last 2 wk), maternal age, ethnicity, education, intelligence, and depression, intervention group, and duration of intervention prior to conception. Infant and young child feeding practices included early initiation of breastfeeding, exclusive breastfeeding at 6 mo, and dietary diversity at 12 mo and 24 mo using the standard WHO/UNICEF indicators [37]. Maternal education was categorized into 4 groups: primary school (completed 1–5 y), secondary school (6–9 y), high school (10–12 y), and college or higher. Maternal depression was measured at baseline using the Center for Epidemiologic Studies Depression Scale (CES-D) [38]. Maternal intellectual ability was assessed using Raven’s Progressive Matrices IQ Test [39] at the time of enrollment (preconception). The intervention group included the FA, IFA, and MM groups. At household level, the quality of the learning environment at home was measured using the infant/toddler Home Observation for Measurement of the Environment (HOME) inventory at 12 mo [40]; the HOME assesses the quality and quantity of the social, emotional, and cognitive support available to a child in the home environment. Household socioeconomic status (SES) index was calculated using principal component analysis of housing quality and assets that were assessed at baseline; the first component derived from component scores was used to divide household SES into tertiles [41,42].

### Statistical analysis

The Kolmogorov-Smirnov test was used for testing the normality of the continuous outcome variables. Descriptive statistics were used to report the characteristics of the study population, with frequencies and percentages to describe categorical variables, means, and SDs to describe quantitative variables. Multivariable linear regressions (for continuous outcome) and logistic regression (for binary outcomes) were used to assess the associations between preconception Hb concentration or anemia on birth outcomes, offspring Hb concentration during the first 1000 d, and child development at 12 mo, 24 mo and 6–7 y. Associations were examined as follows: (1) unadjusted; and (2) adjusted for maternal age, ethnicity, education, SES, infant age and sex, intervention group and duration of time prior to conception, infant and young child feeding practices and child illness. Further adjustment for home environment, mother’s IQ, and depression were made in the models with child development at 12 mo and 24 mo and child intellectual development at 6–7 y as the outcomes. Effect modification by the intervention group was evaluated by including interaction terms in all models. In addition, effect modification by home environment was also evaluated by including interaction terms with maternal Hb concentration for models with child development outcomes. All analyses were conducted using Stata v17 (StataCorp, College Station, TX, USA). The significance level was adjusted for multiple testing using a Bonferroni adjustment at each domain level, including a *P* value of 0.01 for birth outcomes (0.05/5 outcomes), 0.013 for Hb concentration (0.05/4 outcomes), 0.017 for development at 12 and 24 mo (0.05/3 outcomes), and 0.01 at 6–7 y (0.05/5 outcomes).

## Ethical approval

The study was approved by the Ethical Committee of Institute of Social and Medicine Studies in Vietnam and Emory University's Institutional Review Board, Atlanta, Georgia, USA. The trial was registered in the US Clinical Trials registry (identification number NCT01665378). Written informed consent was obtained from all study participants.

## Results

The final analytical sample included 1579 mother-infant pairs women with singleton, live births, and preconception Hb concentration data (Supplemental Figure 1). Selected maternal characteristics at preconception enrollment are shown in Table 1. The mean maternal age was ∼26 y, and nearly half of the sample belonged to minority ethnic groups. Almost all women had ≥1 child at enrollment, and 80% of them were farmers. Approximately one-third of mothers had a low BMI, and 20% were anemic. As described in Table 2, 10% of children were born preterm, 5% with low birth weight, and 16% were SGA. Child anemia affected 62% of children at 3 mo, 66% at 6 mo, 53% at 12 mo, and 42% at 24 mo.

**TABLE 1:** Baseline maternal characteristics at preconception enrollment and child characteristics from birth to 6–7 y^1^

Variable	Total (*n* = 1579)
**Maternal characteristics at preconception enrollment**	
Age, *y*	25.9 ± 4.3
Minority ethnic, %	778 (49.3)
Education level, %	
Primary school	129 (8.2)
Secondary school	854 (54.1)
High school	400 (25.3)
College or higher	195 (12.4)
Work as farmers, %	1258 (79.7)
Number of children ≥ 1, %	1261 (94.6)
BMI, kg/m^2^	19.6 ± 2.0
Low BMI (<18.5), %	485 (30.9)
Hb, g/dl	12.9 ± 1.3
Anemia, % (Hb < 12 g/dL)	313 (19.9)
Mild anemia, % (11–11.9 g/dL)	215 (13.7)
Moderate anemia, % (8–10.9 g/dL)	98 (6.2)
Duration of time prior to conception, wk	28.5 ± 24.8
**Child characteristics at birth**	
Female, %	781 (49.5)
**Infant and young child feeding practices**	
Early initiation of breastfeeding, %	768 (51.4)
Exclusive breastfeeding at 6 mo, %	816 (59.1)
Minimum dietary diversity at 12 mo, %	1060 (82.8)
Minimum dietary diversity at 24 mo, %	851 (70.6)
**Child illness**	
At 3 mo	
Fever, %	281 (20.4)
Diarrhea, %	155 (11.2)
At 6 mo	
Fever, %	359 (28.1)
Diarrhea, %	187 (14.6)
At 12 mo	
Fever, %	503 (39.3)
Diarrhea, %	174 (13.6)
At 24 mo	
Fever, %	466 (32.7)
Diarrhea, %	83 (5.8)
At 6–7 y	
Fever, %	177 (12.7)
Diarrhea, %	16 (1.2)

Hb, hemoglobin. ^1^Values are means (SDs) or n (%).

**TABLE 2:** Child health and development outcomes from birth to 6**–**7 y^1^

Variable	Total (*n* = 1579)
**Child birth outcomes**	
Gestational age, wk	39.2 ± 2.0
Preterm, %	155 (9.9)
Birth weight, g	3083.9 ± 440.3
Low birth weight, %	73 (4.7)
SGA, %	227 (15.5)
Birth length, cm	49.0 ± 3.0
**Child Hb**	
Hb at 3 mo, g/dl	10.6 ± 1.3
Anemia, % (Hb < 11 g/dL)	820 (61.7)
Hb at 6 mo, g/dl	10.5 ± 1.1
Anemia, % (Hb < 11 g/dL)	812 (65.6)
Hb at 12 mo, g/dl	10.8 ± 1.2
Anemia, % (Hb < 11 g/dL)	648 (53.2)
Hb at 24 mo, g/dl	11.2 ± 1.1
Anemia, % (Hb < 11 g/dL)	558 (41.5)
**Bayley scales for infant development at 12 mo**	
Cognitive	112.2 ± 10.3
Language	97.6 ± 11.0
Motor	102.9 ± 11.5
**Bayley scales for infant development at 24 mo**	
Cognitive	99.6 ± 9.9
Language	102.3 ± 10.9
Motor	105.7 ± 11.8
**Wechsler scale global intelligence, performance, and verbal scores at 6–7 y**	
Verbal comprehension index (VCI)	81.8 ± 12.4
Perceptual reasoning index (PRI)	93.1 ± 14.5
Working memory index (WMI)	101.8 ± 11.5
Processing speed index (PSI)	89.4 ± 12.3
Full-scale IQ (FSIQ)	88.3 ± 12.1

Hb, hemoglobin; SGA, small for gestational age. ^1^Values are means (SDs) or n (%).

There were no significant interactions with the intervention group, so pooled findings for all models are presented. Neither preconception Hb concentration nor anemia were significantly associated with birth outcomes (Table 3). However, the lower Hb concentration cut-off for anemia (Hb < 10.81 g/dL) was significantly associated with nearly 2 times risk of SGA (OR 1.83; 95% CI: 1.05, 3.21), Supplemental Table 1. Maternal preconception Hb concentration was positively associated with child Hb concentration at 3 mo (β = 0.06; 95% CI: 0.01, 0.12), 6 mo (0.08; 95% CI:0.03, 0.13), 12 mo (0.10; 95% CI: 0.04–0.15), and 24 mo (0.07; 95% CI: 0.02–0.12) (Table 4). Likewise, a 1 g/dL increase in maternal preconception Hb was associated with an 11%–19% reduced risk of child anemia at 6 mo (0.89; 95% CI: 0.81–0.98), 12 mo (0.81; 95% CI: 0.74–0.89), and 24 mo (0.87; 95% CI: 0.79–0.95). Children born to women who were anemic at preconception were also at higher risk of being anemic 12 mo (OR 1.48; 95% CI: 1.108–2.03) and 24 mo (OR 1.72; 95% CI: 1.26–2.36). These results remained statistically significant when using a more stringent *P* value that accounted for multiple testing except for child Hb concentration at 3 mo and child anemia at 6 mo.

**TABLE 3:** Maternal preconception hemoglobin (Hb) concentration and anemia status and association with birth outcomes

Variable	Birth weight, g^1^		Birth length, cm^1^		Preterm^2^		Gestational Age, wk^1^		Small for gestational age^2^	
	Unadjusted	Adjusted^3^	Unadjusted	Adjusted^3^	Unadjusted	Adjusted^3^	Unadjusted	Adjusted^3^	Unadjusted	Adjusted^3^
Maternal Hb g/dL	9.43 [−6.84, 25.69]	7.48 [−8.95, 23.92]	−0.09 [−0.21, 0.03]	−0.08 [−0.21, 0.04]	1.04 [0.92, 1.18]	1.03 [0.91, 1.17]	−0.02 [−0.10, 0.05]	−0.01 [−0.09, 0.06]	0.91 [0.82, 1.01]	0.93 [0.83, 1.03]
Maternal any anemia (Hb < 12 g/dL)	−32.58 [−87.08, 21.92]	−25.99 [−80.64, 28.66]	−0.06 [−0.45, 0.34]	−0.03 [−0.43, 0.37]	1.14 [0.76, 1.71]	1.16 [0.77, 1.75]	−0.04 [−0.29, 0.21]	−0.06 [−0.31, 0.19]	1.29 [0.92, 1.81]	1.24 [0.88, 1.74]
*n*	1562	1538	1383	1364	1551	1547	1551	1547	1459	1455

1 Values are β [95% CI].

2 Values are OR [95% CI].

3 Adjusted for maternal age, minority status, education, socioeconomic status, infant sex, intervention group, and duration of time prior to conception

**TABLE 4:** Association between preconception maternal and offspring hemoglobin (Hb) concentration and anemia status during the first 1000 d

Variable	Child Hb^1^							
	Hb 3 mo (g/dL)		Hb 6 mo (g/dL)		Hb 12 mo (g/dL)		Hb 24 mo (g/dL)	
	Unadjusted	Adjusted^3^	Unadjusted	Adjusted^4^	Unadjusted	Adjusted^5^	Unadjusted	Adjusted^6^
Maternal Hb (g/dL)	0.06∗ [0.00, 0.11]	0.06∗ [0.01, 0.12]	0.09∗∗ [0.04, 0.13]	0.08∗∗ [0.03, 0.13]	0.11∗∗ [0.06, 0.16]	0.10∗∗ [0.04, 0.15]	0.08∗∗ [0.03, 0.12]	0.07∗∗ [0.02, 0.12]
Any anemia (Hb < 12 g/dL)	−0.16 [−0.34, 0.02]	−0.14 [−0.32, 0.04]	−0.17∗ [−0.33, −0.00]	−0.12 [−0.30, 0.05]	−0.25∗∗ [−0.41, −0.08]	−0.17 [−0.35, 0.01]	−0.24∗∗ [−0.38, −0.09]	−0.24^∗∗^ [−0.42, −0.07]
*n*	1326	1274	1234	1067	1215	1057	1338	1004

Variable	Child Anemia^2^							
	Anemia 3 mo		Anemia 6 mo		Anemia 12 mo		Anemia 24 mo	
	Unadjusted	Adjusted^3^	Unadjusted	Adjusted^4^	Unadjusted	Adjusted^5^	Unadjusted	Adjusted^6^
Maternal Hb (g/dL)	0.92 [0.85, 1.00]	0.93 [0.85, 1.01]	0.89∗∗ [0.81, 0.97]	0.89∗ [0.81, 0.98]	0.82∗∗ [0.75, 0.89]	0.81∗∗ [0.74, 0.89]	0.87∗∗ [0.80, 0.94]	0.87∗∗ [0.79, 0.95]
Maternal any anemia (Hb < 12 g/dL)	1.37∗ [1.03, 1.83]	1.30 [0.97, 1.74]	1.10 [0.81, 1.50]	1.03 [0.75, 1.44]	1.54∗∗ [1.15, 2.05]	1.48∗ [1.08, 2.03]	1.68∗∗ [1.29, 2.19]	1.72∗∗ [1.26, 2.36]
*n*	1326	1274	1234	1067	1215	1057	1338	1004

1 Values are β [95% CI]; ∗*P* < 0.05; ∗∗*P* < 0.013.

2 Values are OR [95% CI]; ∗*P* < 0.05; ∗∗*P* < 0.013.

3 Adjusted for maternal age, minority status, education, socioeconomic status, infant sex, child age, intervention group, duration of time prior to conception, early initiation of breastfeeding, and illness symptoms in the last 2 wk at 3 mo.

4 Adjusted for maternal age, minority status, education, socioeconomic status, infant sex, child age, intervention group, duration of time prior to conception, early initiation of breastfeeding, exclusive breastfeeding at 6 mo, illness symptoms in the last 2 wk at 6 mo.

5 Adjusted for maternal age, minority status, education, socioeconomic status, infant sex, child age, intervention group, duration of time prior to conception, early initiation of breastfeeding, exclusive breastfeeding at 6 mo, minimum dietary diversity at 12 mo, and illness symptoms in the last 2 wk at 12 mo.

6 Adjusted for maternal age, minority status, education, socioeconomic status, infant sex, child age, intervention group, duration of time prior to conception, early initiation of breastfeeding, exclusive breastfeeding at 6 mo, minimum dietary diversity at 24 mo, and illness symptoms in the last 2 wk at 24 mo.

Maternal preconception Hb concentration was positively associated with child motor development at 12 mo in unadjusted models (0.70; 95% CI: 0.23, 1.17), with significant attenuation in the fully adjusted model (0.49; 95% CI: 0.0, 0.99), Table 5. There was, however, evidence of effect modification by home environment, where maternal preconception Hb concentration was significantly associated with child motor development in low home environments (1.16; 95% CI: 0.23, 2.09), Supplemental Table 2. Maternal preconception anemia was negatively associated with child cognition (−1.64; 95% CI: −3.09, −0.19) and language development (−1.61; 95% CI: −3.20, −0.03) at 24 mo, Table 5. However, these associations at 24 mo were not statistically significant when a more conservative *P* value was utilized to take into account multiple testing. Although there were no significant interactions with home environment and child cognitive development, stronger associations with maternal preconception anemia and language development were noted in high home environments at 24 mo (−4.57; 95% CI: −7.67, −1.49) compared with those in a low home environment (−2.24; 95% CI: −4.64,0.17). Maternal preconception Hb and anemia were not significantly associated with child cognition at age 6–7 y (Table 6).

**TABLE 5:** Association between preconception maternal hemoglobin (Hb) concentration and anemia with child development at 12 and 24 mo^1^

Variable	Child development—12 mo					
	Cognition		Language		Motor	
	Unadjusted	Adjusted^2^	Unadjusted	Adjusted^2^	Unadjusted	Adjusted^2^
Maternal Hb (g/dL)	0.44∗ [0.02, 0.86]	0.28 [−0.18, 0.74]	0.21 [−0.24, 0.66]	0.05 [−0.43, 0.53]	0.70^∗∗^ [0.23, 1.17]	0.49 [−0.00, 0.99]
Maternal any anemia (Hb < 12 g/dL)	−0.67 [−2.08, 0.74]	−0.33 [−1.87, 1.20]	−0.76 [−2.26, 0.74]	0.13 [−1.49, 1.75]	−2.17^∗∗^ [−3.74, −0.60]	−1.33 [−3.01, 0.34]
*n*	1295	1060	1294	1061	1293	1060

Variable	Child development—24 mo					
	Cognition		Language		Motor	
	Unadjusted	Adjusted^3^	Unadjusted	Adjusted^3^	Unadjusted	Adjusted^3^
Maternal Hb (g/dL)	0.32 [−0.06, 0.70]	0.33 [−0.10, 0.76]	0.29 [−0.12, 0.71]	0.28 [−0.19, 0.76]	0.25 [−0.21, 0.70]	0.49 [−0.05, 1.03]
Maternal any anemia (Hb < 12 g/dL)	−1.46∗ [−2.73, −0.19]	−1.64^∗^ [−3.09, −0.19]	−1.64∗ [−3.03, −0.26]	−1.61^∗^ [−3.20, −0.03]	−1.13 [−2.66, 0.39]	−1.79 [−3.61, 0.02]
*n*	1407	1044	1404	1044	1403	1043

1 Values are β [95% CI]; ∗*P* < 0.05, ∗∗*P* < 0.017.

2 Adjusted for maternal age, minority status, education, socioeconomic status, infant sex, child age, intervention group, duration of time prior to conception, home environment, maternal IQ, depression, early initiation of breastfeeding, exclusive breastfeeding at 6 mo, minimum dietary diversity at 12 mo, and child illness in the last 2 wk at 12 mo.

3 Adjusted for maternal age, minority status, education, socioeconomic status, infant sex, child age, intervention group, duration of time prior to conception, home environment, maternal IQ, depression, early initiation of breastfeeding, exclusive breastfeeding at 6 mo, minimum dietary diversity at 24 mo, and child illness in the last 2 wk at 24 mo.

**TABLE 6:** Association between preconception maternal hemoglobin (Hb) concentration and anemia with child development at 6**–**7 y^1^

Variable	VCI		PRI		WMI		PSI		*FSIQ*	
	Unadjusted	Adjusted^2^	Unadjusted	Adjusted^2^	Unadjusted	Adjusted^2^	Unadjusted	Adjusted^2^	Unadjusted	Adjusted^2^
Maternal Hb (g/dL)	0.15 [−0.36, 0.67]	−0.05 [−0.65, 0.55]	0.44 [−0.16, 1.03]	−0.01 [−0.69, 0.68]	0.14 [−0.34, 0.62]	0.11 [−0.44, 0.64]	0.44 [−0.06, 0.95]	0.38 [−0.21, 0.97]	0.36 [−0.15, 0.86]	0.10 [−0.47, 0.67]
Maternal any anemia (Hb < 12 g/dL)	−0.63 [−2.36, 1.10]	−0.19 [−2.20, 1.82]	−2.03∗ [−4.03, −0.03]	−0.48 [−2.79, 1.83]	−1.55 [−3.14, 0.04]	−1.27 [−3.08, 0.55]	−1.74∗ [−3.44, −0.04]	−1.31 [−3.29, 0.68]	−1.80∗ [−3.49, −0.12]	−0.95 [−2.87, 0.98]
N	1266	904	1266	904	1263	902	1266	904	1263	902

FSIQ, full-scale intelligence quotient; PRI, perceptual reasoning index; PSI, processing speed index; VCI, verbal comprehension index; WMI, working memory index;.

1 Values are β [95% CI], ∗*P* < 0.05, ∗∗*P* < 0.01.

2 Adjusted for maternal age, minority status, education, socioeconomic status, infant sex, child age intervention group, duration of time prior to conception, home environment, maternal intelligence quotient, depression, early initiation of breastfeeding, exclusive breastfeeding at 6 mo, minimum dietary diversity at 24 mo, and child illness in the last 2 wk at 6–7 y.

## Discussion

We find that maternal preconception Hb concentration was positively associated with offspring Hb concentrations during the first 2 y of life. In contrast, associations between maternal preconception Hb and anemia and child development during 1^st^ 1000 d were weak or mixed. Preconception Hb concentration was also not a significant predictor of birth outcomes or cognition at 6–7 y after adjustment for maternal and sociodemographic variables in our study population.

Our null findings on maternal preconception Hb concentration and birth outcomes contrast with earlier findings in a systematic review where preconception anemia was associated with an increased risk of low birthweight (OR 1.72; 95% CI: 1.31–2.26) and SGA births (OR 1.79; 95% CI: 1.39–2.31) [5]. However, the findings on the lack of associations with preterm birth are consistent with the systematic review. There are several potential reasons for these differences. First, in our cohort, the prevalence of low birthweight was very low (<5%). Another factor may be the prevalence, severity, and etiology of anemia in our context. The overall prevalence of preconception anemia was 20%, and the majority of anemia was mild (Hb concentration 11.0–11.9 g/dL). Recently there have been calls to revisit global guidelines and re-examine WHO Hb concentration thresholds [43], and recent multicountry studies in nonpregnant women of reproductive age [36] and pregnant women [44] have suggested current cutoffs may be too high. It may be that lower thresholds to define anemia may be more sensitive for detecting relationships with adverse outcomes. To examine this, we ran an exploratory posthoc analysis using a lower Hb concentration cut-off (Hb < 10.81g/dL) as suggested by the Addo et al. [36] article with nonpregnant women. Although most results remained consistent, the lower Hb concentration cut-off was more sensitive for detecting associations with SGA. Further research is needed to understand optimal preconception Hb concentration cutoffs for birth outcomes. In addition, high compliance (>80%) with preconception and pregnancy supplementation may contribute to our null findings. Another potential mechanism could be the etiology of anemia. In our population, the prevalence of iron deficiency was quite low, with only 14% of women with low iron stores (ferritin < 30 μg/L) and <5% with iron deficiency (ferritin < 12 μg/L or transferrin receptor > 8.3 mg/L) [35]. Prior research in this population has indicated there may be other important driving factors for anemia, including hookworm infections and potentially hemoglobinopathies [45]. Although data on thalassemia or other heredity hematologic diseases were not directly available for the study participants, the previously reported associations between Hb concentration and ethnicity and prevalence of hemoglobinopathies in the region suggest this may be an important consideration [[45], [46], [47], [48]]. The role of the etiology of anemia and its association with birth outcomes remains unclear and an important research gap [5].

In our cohort, we present novel data on the association of maternal preconception Hb concentration and child Hb concentration across the first 2 y of life. Women who are anemic and/or iron deficient before conception are also at increased risk of getting worse during pregnancy because of the high physiologic demands of pregnancy [49]. Maternal anemia during pregnancy can also impact the offspring's iron stores at birth [50,51], which in turn can affect the offspring’s risk of developing anemia during infancy and early childhood [[52], [53], [54]]. Our results are in alignment with existing literature on prospective studies of maternal anemia during pregnancy and child anemia as well as cross-sectional analyses of associations between maternal and child anemia in early childhood [[55], [56], [57]].

We report significant associations with maternal preconception Hb concentration on child motor development in the first y of life, particularly in low home environments, but no long-term associations at 24 mo. We also report associations between maternal preconception anemia and child cognition and language development at 24 mo, which were stronger in high home environments for the latter. The rationale for mixed findings on home environment is unclear and merits further examination. It is possible this is because of different mechanisms, as the role of stimulation may vary by child age and outcomes, but it could also be due to chance and recommend caution in interpterion given multiple testing. Periconceptional nutrition may influence early childhood development by affecting the growth and development of key organs, such as the brain, liver, and pancreas, during the first few weeks of pregnancy [58]. However, very few studies have examined maternal status preconception or the long-term impact of maternal anemia on functional outcomes beyond birth, such as child IQ or school readiness [59,60]. In a Swedish cohort of over 500,000 children, maternal anemia in early pregnancy was associated with over a twofold increased risk of development of intellectual disability in the offspring between the ages of 6–29 y, but much weaker associations were seen for anemia in late pregnancy [61]. In the larger PRECONCEPT trial, children born to women in the iron and folic acid preconception supplementation group had improved motor development, especially fine motor development (IFA compared with FA: 0.41; 95% CI: 0.05, 0.77), but there were no significant differences in mental or language scores at age 2 y [23]. At 6–7 y, children born to women in the MM group, compared with the FA group, had higher FSIQ (β = 1.7; 95% CI: 0.1, 3.3), WMI (β = 1.7; 95% CI: 0.2, 3.2), and PSI (β = 2.5; 95% CI: 0.9, 4.1) [24]. Fetal brain development begins early in pregnancy, with most structural features of the brain appearing in the first 8 wk following conception [62,63]. There are critical windows of opportunity where health, nutrition, security and safety, responsive caregiving, and early learning play crucial roles in allowing children to reach their full potential [64]. Maternal nutrition and the availability of key micronutrients are required to support optimal brain development [65,66]. The preconception time period may be particularly important as many women may not seek antennal care until the second trimester of pregnancy, when much of the early development has already taken place.

Our study provides novel insight into the importance of maternal anemia during the preconception period and child health and development. This is one the largest prospective studies to date to examine the role of maternal preconception nutrition on long-term child health outcomes. A key strength of this work is the prospective design and the low rates of attrition (<10%) over the past decade. Our design and high-quality data collection allowed for accurate estimation of gestational age and, thus, calculation of key birth outcome variables such as preterm birth and SGA [67]. However, there are also a number of important limitations to consider. It is possible significant associations are due to chance and a statistical artifact. In particular, we recommend a conservative interpretation of the findings with child development, given the mixed findings. It would be beneficial for future work to examine the quality of the learning and nurturing environment prospectively across early childhood. Further examination of the etiology of anemia would be valuable to better understand the underlying biological mechanisms of the associations. The limited sample size of women with iron deficiency anemia or moderate/severe anemia precludes us from further examination of the role of etiology of anemia or Hb concentration cutoffs on child health and development. Furthermore, our study may have limited generalizability given the overall high preconception and pregnancy supplementation in the context of an ongoing research study. Our analysis is observational in nature and thus cannot establish causality.

In summary, maternal preconception Hb concentration was associated with child Hb concentrations across the first 1000 d. Modest associations with maternal preconception Hb concentration and anemia and child development in early life merit further examination. However, in this cohort from Vietnam, preconception Hb concentration was not a significant predictor of birth outcomes or long-term child development. Our findings support the need to revisit the use of measuring Hb concentration in women of reproductive age to predict later risk of adverse outcomes, and more research is needed in settings with a greater burden of anemia and iron deficiency as we support programs and policies to expand maternal anemia reduction programs to the adolescent and preconception periods.

## Data Availability

Data described in the manuscript, code book, and analytic code will be made available upon request, pending approval by the study team.

## References

[1] Stevens G.A., Paciorek C.J., Flores-Urrutia M.C., Borghi E., Namaste S., Wirth J.P. (2022). National, regional, and global estimates of anaemia by severity in women and children for 2000–19: a pooled analysis of population-representative data. Lancet Glob. Health..

[2] Leroy J.L., Frongillo E.A., Dewan P., Black M.M., Waterland R.A. (2020). Can children catch up from the consequences of undernourishment? Evidence from child linear growth, developmental epigenetics, and brain and neurocognitive development. Adv. Nutr..

[3] Black R.E., Victora C.G., Walker S.P., Bhutta Z.A., Christian P., de Onis M. (2013). Maternal and child undernutrition and overweight in low-income and middle-income countries. Lancet.

[4] Chaparro C.M., Suchdev P.S. (2019). Anemia epidemiology, pathophysiology, and etiology in low- and middle-income countries. Ann. N. Y. Acad. Sci..

[5] Young M.F., Oaks B.M., Tandon S., Martorell R., Dewey K.G., Wendt A.S. (2019). Maternal hemoglobin concentrations across pregnancy and maternal and child health: a systematic review and meta-analysis. Ann. N. Y. Acad. Sci..

[6] Grantham-McGregor S., Cheung Y.B., Cueto S., Glewwe P., Richter L., Strupp B. (2007). Developmental potential in the first 5 years for children in developing countries. Lancet.

[7] Walker S.P., Wachs T.D., Gardner J.M., Lozoff B., Wasserman G.A., Pollitt E. (2007). Child development: risk factors for adverse outcomes in developing countries. Lancet.

[8] Lozoff B., Wolf A.W., Jimenez E. (1996). Iron-deficiency anemia and infant development: effects of extended oral iron therapy. J. Pediatr..

[9] Algarín C., Nelson C.A., Peirano P., Westerlund A., Reyes S., Lozoff B. (2013). Iron-deficiency anemia in infancy and poorer cognitive inhibitory control at age 10 years. Dev. Med. Child. Neurol..

[10] Menon K.C., Ferguson E.L., Thomson C.D., Gray A.R., Zodpey S., Saraf A. (2016). Effects of anemia at different stages of gestation on infant outcomes. Nutrition.

[11] Dean S.V., Lassi Z.S., Imam A.M., Bhutta Z.A. (2014). Preconception care: nutritional risks and interventions. Reprod. Health..

[12] King J.C. (2016). A summary of pathways or mechanisms linking preconception maternal nutrition with birth outcomes. J. Nutr..

[13] Ramakrishnan U., Grant F., Goldenberg T., Zongrone A., Martorell R. (2012). Effect of women's nutrition before and during early pregnancy on maternal and infant outcomes: a systematic review. Paediatr. Perinat. Epidemiol..

[14] Young M.F., Nguyen P.H., Addo O.Y., Hao W., Nguyen H., Pham H. (2015). The relative influence of maternal nutritional status before and during pregnancy on birth outcomes in Vietnam. Eur. J. Obstet. Gynecol. Reprod. Biol..

[15] Fleming T.P., Watkins A.J., Velazquez M.A., Mathers J.C., Prentice A.M., Stephenson J. (2018). Origins of lifetime health around the time of conception: causes and consequences. Lancet.

[16] Maghsoudlou S., Cnattingius S., Stephansson O., Aarabi M., Semnani S., Montgomery S.M. (2016). Maternal haemoglobin concentrations before and during pregnancy and stillbirth risk: a population-based case-control study. B.M.C. Pregnancy Childbirth..

[17] Yi S.W., Han Y.J., Ohrr H. (2013). Anemia before pregnancy and risk of preterm birth, low birth weight and small-for-gestational-age birth in Korean women. Eur. J. Clin. Nutr..

[18] Ronnenberg A.G., Wood R.J., Wang X., Xing H., Chen C., Chen D. (2004). Preconception hemoglobin and ferritin concentrations are associated with pregnancy outcome in a prospective cohort of Chinese women. J. Nutr..

[19] Zhang X., Xu Q., Yang Y., Wang L., Liu F., Li Q. (2018). Preconception Hb concentration and risk of preterm birth in over 2.7 million Chinese women aged 20–49 years: a population-based cohort study. Br. J. Nutr..

[20] Larson L.M., Kubes J.N., Ramírez-Luzuriaga M.J., Khishen S., Shankar A.H., Prado E.L. (2019). Effects of increased hemoglobin on child growth, development, and disease: a systematic review and meta-analysis. Ann. N. Y. Acad. Sci..

[21] Nguyen P.H., Lowe A.E., Martorell R., Nguyen H., Pham H., Nguyen S. (2012). Rationale, design, methodology and sample characteristics for the Vietnam pre-conceptual micronutrient supplementation trial (PRECONCEPT): a randomized controlled study. B.M.C. Public Health..

[22] Ramakrishnan U., Nguyen P.H., Gonzalez-Casanova I., Pham H., Hao W., Nguyen H. (2016). Neither preconceptional weekly multiple micronutrient nor iron-folic acid supplements affect birth size and gestational age compared with a folic acid supplement alone in rural Vietnamese women: a randomized controlled trial. J. Nutr..

[23] Nguyen P.H., Gonzalez-Casanova I., Young M.F., Truong T.V., Hoang H., Nguyen H. (2017). Preconception micronutrient supplementation with iron and folic acid compared with folic acid alone affects linear growth and fine motor development at 2 years of age: a randomized controlled trial in Vietnam. J. Nutr..

[24] Nguyen P.H., Young M.F., Tran L.M., Khuong L.Q., Duong T.H., Nguyen H.C. (2021). Preconception micronutrient supplementation positively affects child intellectual functioning at 6 y of age: a randomized controlled trial in Vietnam. Am. J. Clin. Nutr..

[25] Gonzalez-Casanova I., Nguyen P.H., Young M.F., Harding K.B., Reinhart G., Nguyen H. (2017). Predictors of adherence to micronutrient supplementation before and during pregnancy in Vietnam. B.M.C. Public Health..

[26] Gibson R. (2005).

[27] Villar J., Cheikh Ismail L., Victora C.G., Ohuma E.O., Bertino E., Altman D.G. (2014). International standards for newborn weight, length, and head circumference by gestational age and sex: the Newborn Cross-Sectional Study of the INTERGROWTH-21st Project. Lancet.

[28] World Health Organization (2011). https://www.who.int/publications/i/item/WHO-NMH-NHD-MNM-11.1.

[29] Bayley N. (2005).

[30] Tran T.D., Biggs B.A., Tran T., Simpson J.A., Hanieh S., Dwyer T. (2013). Impact on infants’ cognitive development of antenatal exposure to iron deficiency disorder and common mental disorders. PLOS. ONE..

[31] Tran T.D., Tran T., Simpson J.A., Tran H.T., Nguyen T.T., Hanieh S. (2014). Infant motor development in rural Vietnam and intrauterine exposures to anaemia, iron deficiency and common mental disorders: a prospective community-based study. B.M.C. Pregnancy Childbirth..

[32] Wechsler D. (2003).

[33] Dang H.M., Weiss B., Pollack A., Nguyen M.C. (2012). Adaptation of the Wechsler Intelligence Scale for Children-IV (WISC-IV) for Vietnam. Psychol. Stud. (Mysore)..

[34] (2023). Hemocue. Hemocue Hb301.

[35] Nguyen P.H., Young M., Gonzalez-Casanova I., Pham H.Q., Nguyen H., Truong T.V. (2016). Impact of preconception micronutrient supplementation on anemia and iron status during pregnancy and postpartum: a randomized controlled trial in rural Vietnam. PLOS. ONE.

[36] Addo O.Y., Yu E.X., Williams A.M., Young M.F., Sharma A.J., Mei Z. (2021). Evaluation of hemoglobin cutoff levels to define anemia among healthy individuals. JAMA. Netw. Open..

[37] World Health Organization and the United Nations Children’s Fund (UNICEF) (2021). https://www.who.int/publications/i/item/9789240018389.

[38] Radloff L.S. (1977). The CES-D Scale: a self-report depression scale for research in the general population. Appl. Psychol. Meas..

[39] Raven J., Raven J.C., Court J.H. (1998).

[40] Bradley R.H., Caldwell B.M. (1988). Using the home inventory to assess the family environment. Pediatr. Nurs..

[41] Vyas S., Kumaranayake L. (2006). Constructing socio-economic status indices: how to use principal components analysis. Health Policy Plan.

[42] Gwatkin D.R., Rutstein S., Johnson K., Suliman E., Wagstaff A., Amouzou A. (2007). Socio-economic differences in health, nutrition, and population within developing countries: an overview, Niger. J. Clin. Pract..

[43] Garcia-Casal M.N., Pasricha S.R., Sharma A.J., Peña-Rosas J.P. (2019). Use and interpretation of hemoglobin concentrations for assessing anemia status in individuals and populations: results from a WHO technical meeting. Ann. N. Y. Acad. Sci..

[44] Ohuma E.O., Young M.F., Martorell R., Ismail L.C., Peña-Rosas J.P., Purwar M. (2020). International values for haemoglobin distributions in healthy pregnant women. eClinicalMedicine.

[45] Nguyen P.H., Gonzalez-Casanova I., Nguyen H., Pham H., Truong T.V., Nguyen S. (2015). Multicausal etiology of anemia among women of reproductive age in Vietnam. Eur. J. Clin. Nutr..

[46] Anh T.M., Sanchaisuriya K., Kieu G.N., Tien D.N., Thu H.B.T., Sanchaisuriya P. (2019). Thalassemia and hemoglobinopathies in an ethnic minority group in Northern Vietnam. Hemoglobin.

[47] Nguyen N.T., Sanchaisuriya K., Sanchaisuriya P., Van Nguyen H., Phan H.T.T., Fucharoen G. (2017). Thalassemia and hemoglobinopathies in an ethnic minority group in Central Vietnam: implications to health burden and relationship between two ethnic minority groups. J. Community Genet..

[48] Svasti S., Hieu T.M., Munkongdee T., Winichagoon P., Van Be T., Van Binh T. (2002). Molecular analysis of beta-thalassemia in South Vietnam. Am. J. Hematol..

[49] Bothwell T.H. (2000). Iron requirements in pregnancy and strategies to meet them. Am. J. Clin. Nutr..

[50] Shukla A.K., Srivastava S., Verma G. (2019). Effect of maternal anemia on the status of iron stores in infants: a cohort study. J. Family Community Med..

[51] Viteri F.E. (2011). Iron endowment at birth: maternal iron status and other influences. Nutr. Rev..

[52] Georgieff M.K., Wewerka S.W., Nelson C.A., Deregnier R.A. (2002). Iron status at 9 months of infants with low iron stores at birth. J. Pediatr..

[53] Hirata M., Kusakawa I., Ohde S., Yamanaka M., Yoda H. (2017). Risk factors of infant anemia in the perinatal period. Pediatr. Int..

[54] Zhang Y., Jin L., Liu J.M., Ye R., Ren A. (2016). Maternal hemoglobin concentration during gestation and risk of anemia in infancy: secondary analysis of a randomized controlled trial. J. Pediatr..

[55] Engidaye G., Melku M., Yalew A., Getaneh Z., Asrie F., Enawgaw B. (2019). Under nutrition, maternal anemia and household food insecurity are risk factors of anemia among preschool aged children in Menz Gera Midir district, Eastern Amhara, Ethiopia: a community based cross-sectional study. B.M.C. Public Health..

[56] Onyeneho N.G., Corsi D.J., Kurpad A., Subramanian S.V. (2019). Intergenerational influences on childhood anaemia. Matern. Child. Nutr..

[57] Heesemann E., Mähler C., Subramanyam M.A., Vollmer S. (2021). Pregnancy anaemia, child health and development: a cohort study in rural India. B.M.J. Open..

[58] Cetin I., Berti C., Calabrese S. (2010). Role of micronutrients in the periconceptional period. Hum. Reprod. Update..

[59] Drassinower D., Lavery J.A., Friedman A.M., Levin H.I., Običan S.G., Ananth C.V. (2016). The effect of maternal haematocrit on offspring IQ at 4 and 7 years of age: a secondary analysis. B.J.O.G..

[60] Leonard H., de Klerk N., Bourke J., Bower C. (2006). Maternal health in pregnancy and intellectual disability in the offspring: a population-based study. Ann. Epidemiol..

[61] Wiegersma A.M., Dalman C., Lee B.K., Karlsson H., Gardner R.M. (2019). Association of prenatal maternal anemia with neurodevelopmental disorders. JAMA Psychiatry.

[62] O’rahilly R., Müller F. (2008). Significant features in the early prenatal development of the human brain. Ann. Anat..

[63] Marsh R., Gerber A.J., Peterson B.S. (2008). Neuroimaging studies of normal brain development and their relevance for understanding childhood neuropsychiatric disorders. J. Am. Acad. Child Adolesc. Psychiatry..

[64] Black M.M., Walker S.P., Fernald L.C.H., Andersen C.T., DiGirolamo A.M., Lu C. (2017). Early childhood development coming of age: science through the life course. Lancet.

[65] Stephenson J., Heslehurst N., Hall J., Schoenaker D.A.J.M., Hutchinson J., Cade J.E. (2018). Before the beginning: nutrition and lifestyle in the preconception period and its importance for future health. Lancet.

[66] Georgieff M.K. (2008). The role of iron in neurodevelopment: fetal iron deficiency and the developing hippocampus. Biochem. Soc. Trans..

[67] Deputy N.P., Nguyen P.H., Pham H., Nguyen S., Neufeld L., Martorell R. (2017). Validity of gestational age estimates by last menstrual period and neonatal examination compared to ultrasound in Vietnam, B.M.C. Pregnancy Childbirth.

