# Supplementary material for: Long-Term Association Between Maternal Preconception Hemoglobin Concentration, Anemia, and Child Health and Development in Vietnam
Source: J Nutr. 2023 Mar 15;153(5):1597–606. doi: 10.1016/j.tjnut.2023.03.015 (PMC10367189; doi:10.1016/j.tjnut.2023.03.015)
Supplement: Multimedia component 1 [file mmc1.docx]

# Long term association between of maternal preconception hemoglobin and anemia and child health and development in Vietnam. Young MF et. al.,

**Supplementary Figure 1: Participant flow diagram**

**Supplementary Table 1. Maternal preconception Hb < 10.81 g/dL and association with birth outcomes**^1^

| **Variable** | **Birth weight^1^** | | **Birth length^1^** | | **Preterm^2^** | | **Gestational age^1^** | | **Small for gestational age^2^** | |
| --- | --- | --- | --- | --- | --- | --- | --- | --- | --- | --- |
|  | Unadjusted | Adjusted^3^ | Unadjusted | Adjusted^3^ | Unadjusted | Adjusted^3^ | Unadjusted | Adjusted^3^ | Unadjusted | Adjusted^3^ |
| Maternal any anemia  (Hb< 10.81 g/dL) | -80.95 [-179.2,17.3] | -75.89 [-174.3,22.6] | -0.09 [-0.8,0.6] | -0.16 [-0.9,0.56] | 1.33 [0.7,2.7] | 1.40 [0.7,2.8] | -0.31 [-0.,0.14] | -0.34 [-0.8,0.1] | 1.85 [1.07,3.22] | 1.83 [1.05,3.21] |
| N | 1562 | 1558 | 1383 | 1379 | 1551 | 1547 | 1551 | 1547 | 1459 | 1455 |

^1^Values are β [95%CI])

^2^Values are OR [95%CI])

^3^ Adjusted for maternal age, minority status, education, SES, infant sex, intervention group

**Supplementary Table 2. Association between preconception maternal hemoglobin and anemia with motor development at 12 mo, by home environment**

|  | **Motor Development – 12 months** | | | | | |
| --- | --- | --- | --- | --- | --- | --- |
|  | **Low home environment** | | **Medium home environment** | | **High home environment** | |
| **Variable** | **Unadjusted** | **Adjusted**^2^ | **Unadjusted** | **Adjusted**^2^ | **Unadjusted** | **Adjusted**^2^ |
| Maternal Hb | 1.47^**^ [0.61,2.32] | 1.16** [0.23,2.09] | 0.38 [-0.34,1.09] | 0.43 [-0.33,1.18] | 0.22 [-0.66,1.10] | -0.31 [-1.29,0.67] |
|  |  |  |  |  |  |  |
| Maternal any anemia (Hb < 12 g/dL) | -4.67^**^ [-7.38,-1.97] | -2.94 [-5.97,0.08] | -2.73^*^ [-5.17,-0.28] | -2.31 [-4.96,0.35] | 2.00 [-1.03,5.04] | 2.68 [-0.59,5.96] |
| N | 377 | 297 | 543 | 463 | 368 | 300 |
|  | **Cognitive Development - 24 months** | | | | | |
|  | **Low home environment** | | **Medium home environment** | | **High home environment** | |
| **Variable** | **Unadjusted** | **Adjusted**^2^ | **Unadjusted** | **Adjusted**^2^ | **Unadjusted** | **Adjusted**^2^ |
| Maternal Hb | 0.44 [-0.22,1.11] | 0.09 [-0.68,0.86] | 0.15 [-0.44,0.74] | 0.44 [-0.23,1.11] | 0.36 [-0.40,1.12] | 0.42 [-0.46,1.30] |
|  |  |  |  |  |  |  |
| Maternal any anemia (Hb < 12 g/dL) | -2.50^*^ [-4.59,-0.40] | -1.32 [-3.78,1.14] | -0.13 [-2.14,1.87] | -1.28 [-3.64,1.08] | -2.02 [-4.63,0.59] | -2.38 [-5.32,0.55] |
| N | 423 | 320 | 589 | 445 | 390 | 300 |
|  | **Language Development – 24 months** | | | | | |
|  | **Low home environment** |  | **Medium home environment** |  | **High home environment** |  |
| **Variable** | **Unadjusted** | **Adjusted**^2^ | **Unadjusted** | **Adjusted**^2^ | **Unadjusted** | **Adjusted**^2^ |
| Maternal Hb | 0.50 [-0.26,1.26] | -0.25 [-1.13,0.63] | -0.29 [-0.92,0.33] | 0.17 [-0.57,0.90] | 0.90^*^ [0.07,1.72] | 0.92 [-0.01,1.85] |
|  |  |  |  |  |  |  |
| Maternal any anemia (Hb < 12 g/dL) | -2.24 [-4.64,0.17] | 0.35 [-2.47,3.18] | 0.70 [-1.41,2.81] | -0.85 [-3.44,1.73] | -4.32^**^ [-7.13, -1.52] | -4.57^**^ [-7.67, -1.49] |
| N | 423 | 320 | 589 | 445 | 387 | 279 |

^1^Values are β [95%CI]); *p<0.05, **p<0.017

^2^ Adjusted for maternal age, minority status, education, socioeconomic status, infant sex, child age, intervention group, duration of time prior to conception, home environment, maternal IQ, depression, early initiation of breastfeeding, exclusive breastfeeding at 6 mo, minimum dietary diversity at 12 mo, and child illness in the last 2 weeks at 12 mo

^3^ Adjusted for maternal age, minority status, education, socioeconomic status, infant sex, child age, intervention group, duration of time prior to conception, home environment, maternal IQ, depression, early initiation of breastfeeding, exclusive breastfeeding at 6 mo, minimum dietary diversity at 24 mo, and child illness in the last 2 weeks at 24 mo
